# Functioning of vascular access and satisfaction with nursing care among patients undergoing haemodialysis

**DOI:** 10.1007/s11255-025-04772-z

**Published:** 2025-09-22

**Authors:** Kamil Sikora, Robert Jan Łuczyk, Agnieszka Zwolak, Agnieszka Wawryniuk, Marta Łuczyk

**Affiliations:** 1 Department of Internal Medicine and Internal Nursing, Chair of Preventive Nursing, Faculty of Health Sciences, Medical University of Lublin, Ul. Chodźki 7, 20-093 Lublin, Poland; 2 Department of Long-Term Care Nursing, Chair of Preventive Nursing, Faculty of Health Sciences, Medical University of Lublin, Ul. Chodźki 7, 20-093 Lublin, Poland

**Keywords:** Satisfaction, Renal dialysis, Vascular fistula, Central venous catheter, Patient-reported outcomes, Dialysis nursing

## Abstract

**Background:**

Patient satisfaction is increasingly recognized as an essential indicator of the quality of healthcare, including nursing care, particularly in the context of chronic therapies such as haemodialysis. The condition and functioning of vascular access, crucial for effective renal replacement therapy, may significantly influence patients’ perception of the care received.

**Objective:**

To assess the relationship between the functioning of vascular access and satisfaction with nursing care among patients undergoing haemodialysis.

**Methods:**

This cross-sectional study was conducted between January 2021 and December 2022 among 202 haemodialysis patients in Poland. Data were collected using a proprietary questionnaire assessing satisfaction with nursing care, the Vascular Access Questionnaire (VAQ), and the Visual Analogue Scale (VAS) for pain assesment. The results are summarised in a statistical analysis included descriptive statistics, factor analysis, Cronbach’s alpha, Pearson’s correlation coefficient, and the Shapiro–Wilk test.

**Results:**

The proprietary satisfaction scale showed high internal consistency (Cronbach’s alpha = 0.983) and a single-factor structure. The mean satisfaction score was 3.79 (SD = 1.00), and the mean VAQ score was 13.79 (SD = 11.22). A moderate negative correlation was found between vascular access problems and satisfaction with nursing care (*r* = –0.387; *p* < 0.001). No significant relationship was observed between pain level and satisfaction of nursing care (*r* = –0.091; *p* = 0.215).

**Conclusions:**

Satisfaction with nursing care is inversely related to the severity of vascular access problems in haemodialysis patients. The findings underscore the importance of maintaining well-functioning vascular access and addressing patient concerns as part of comprehensive dialysis care.

## Introduction

One of the elements of evaluating the quality of medical services, including the quality of nursing care, is to measure the patient’s subjective satisfaction with the care provided. This parameter is increasingly being placed on a par with objective health indicators, along with growing patient expectations regarding the medical services provided. Patient satisfaction is defined as the fulfilment of the expectations of a person receiving healthcare, the satisfaction of their needs – both physical and emotional, social and spiritual – or as the difference between the patient’s expectations and what they receive. Irrespective of the definition, patient satisfaction is considered to be a measure of the quality of healthcare, including nursing care. This indicates the necessity for its systematic assessment. In this context, both proprietary tools, adapted to the specific nature of a given healthcare facility, and standardised tools are employed for this purpose. The measurement of patient satisfaction, encompassing satisfaction with nursing care, can serve as a foundation for the development of care standards that encompass clinical effectiveness, patient safety and patient preferences [1–3].

Patient satisfaction with vascular access constitutes a significant factor in determining satisfaction with renal replacement therapy in its entirety, as well as its effectiveness. It is imperative that dialysis centre staff are cognisant of patients’ opinions with regard to their vascular access, in order that they are able to identify any concerns that patients may have, highlight any gaps in education and implement appropriate corrective measures. In the domain of education for haemodialysis patients, the assessment of satisfaction with vascular access can serve as an evaluative metric for the efficacy of implemented measures. A plethora of studies have been conducted to assess patient satisfaction with care during renal replacement therapy. The majority of these studies have yielded a positive assessment of care related to vascular access for haemodialysis [4]. Furthermore, it has been established that satisfaction with care is associated with vascular access, with patients with arteriovenous fistula (AVF) demonstrating a higher level of satisfaction compared to patients with central venous catheter (CVC) [5, 6]. A multitude of studies have been conducted by R.R. Quinn et al., M. Field et al., and S.D. Kosa et al. utilising the vascular access questionnaire (VAQ). These studies have predominantly evaluated the clinical implications of access characteristics, encompassing its type, duration, number of hospitalisations, age, and other pertinent factors. In our own studies, we attempted to observe the correlation between the functioning of vascular access and the perception of pain during the care of this access, as well as satisfaction with care. It is hypothesised that this may have a particular impact on the nursing care of these patients [7–9].

### Objective of the work

The aim of the study was to assess the relationship between vascular access functioning and satisfaction with nursing care among patients undergoing haemodialysis.

## Material and methods

The study was conducted between January 2021 and December 2022. The research sample was selected from among patients undergoing renal replacement therapy using haemodialysis at a dialysis centre located in Lublin, Poland (120 respondents), as well as from among patients associated with an online community of dialysis patients from other centres in Poland (112 respondents). Patients from the online group were included in the study through the distribution of questionnaires via a nationwide association of dialysis patients. A total of 232 participants took part in the main stage of the study, of whom data obtained from 202 respondents were qualified for statistical analysis. The primary study was preceded by a pilot stage conducted in a group of 120 people.

The inclusion criteria for participation in the study were as follows: subjects had to be over the age of 18, diagnosed with chronic kidney disease and undergoing haemodialysis treatment, in addition to having active vascular access that enabled haemodialysis therapy. Voluntary informed consent was also a prerequisite for inclusion. The study design was approved by the Bioethics Committee at the Medical University of Lublin (resolution no. KE-0254/178/2021).

The study employed a diagnostic survey method, utilising techniques such as an auditorium survey, a distributed survey, an online survey, and direct interviews. The following research tools were utilised in the data collection process:

**The original questionnaire** comprised questions regarding the subject’s sociodemographic variables, including age, gender, and the type of vascular access.

**The Vascular Access Questionnaire (VAQ)** is a tool designed for the objective and subjective assessment of factors related to the functioning of vascular access for dialysis therapy. The document under scrutiny contains 17 potential problems experienced by the patient. Each patient is evaluated using a 5-point Likert scale, where 1 indicates that the patient has not experienced any discomfort related to vascular access over the past 4 weeks, and 5 indicates that the problem has caused significant distress. The total score has been shown to be a useful tool in determining the severity of problems related to the functioning of vascular access [7, 8].

**The Visual Analogue Scale (VAS)** is a tool employed to evaluate the pain experienced by patients undergoing vascular access procedures. In the present study, the pain assessment encompassed the previous week.

**The original questionnaire, entitled ‘Satisfaction with Nursing Care at the Dialysis Centre’**, was utilised for the purpose of evaluating patient satisfaction with nursing care. The tool comprises 17 statements delineating the care provided by a dialysis nurse during a patient’s stay at the dialysis station. Patients are invited to rate each statement on a 5-point scale, where 1 = completely dissatisfied and 5 = completely satisfied.

The results obtained were compiled in a statistical analysis using the R programming language version 4.2.2 and the RStudio environment version 2022.12.0. Statistically significant results were defined as those with a p-value less than 0.05. The results were presented using basic statistical measures. The normality of the distribution was verified using the Shapiro–Wilk test, and the correlation analyses between the studied variables were presented based on Pearson’s correlation coefficient r.

## Results

### A pilot study

A pilot study was conducted on a group of 120 patients undergoing renal replacement therapy in order to verify the validity of the proprietary research tool designed to measure satisfaction with nursing care. In order to verify the psychometric values of the proprietary tool, a factor analysis was performed using the principal component method. Its reliability was verified using Cronbach’s alpha coefficient, and its validity was verified by comparing the results obtained with the proprietary tool and the results obtained in the care satisfaction scales of the Kidney Disease and Quality of Life™ Short Form (KDQOL-SF) tool for assessing the quality of life of people with kidney disease. The factor analysis was conducted utilising principal component analysis with Varimax rotation. Table 1 presents statistics describing the results obtained by respondents in each element of the author’s tool.

**Table 1:** Components of the author’s questionnaire – distribution of results obtained by respondents

Questionaire component	M	SD	Me	Min	Max	Skew	Kurt
The efficiency of nurses in performing their work	3.82	0.95	4	2	5	− 0.33	− 0.88
The presence of a nurse nearby when needed	3.86	1.07	4	1	5	− 0.61	− 0.27
The nurses’ knowledge of your illness and care	3.74	1.07	4	1	5	− 0.57	− 0.35
The speed with which nurses responded to your call/alarm	3.97	1.06	4	1	5	− 0.86	− 0.02
The way nurses created a friendly atmosphere during the procedure	3.84	1.1	4	1	5	− 0.7	− 0.3
The amount of information you received from nurses about your health and the scope of care	3.61	1.13	4	1	5	− 0.58	− 0.5
The frequency with which nurses checked to see if you were okay	3.74	1.07	4	1	5	− 0.65	− 0.27
The nurses’ willingness to help	3.99	1.02	4	2	5	− 0.59	− 0.9
The way the nurses explained various issues related to their actions to you	3.78	1.09	4	1	5	− 0.72	− 0.29
Your opinion on how the nurse treated you	3.88	1.07	4	1	5	− 0.75	− 0.24
How the nurses listened to your concerns and worries	3.76	1.08	4	1	5	− 0.64	− 0.3
The nurses’ willingness to fulfil your requests	3.85	1.05	4	1	5	− 0.61	− 0.5
The nurses’ assurance of privacy	3.73	1.12	4	1	5	− 0.68	− 0.23
The nurses’ awareness of your needs	3.74	1.09	4	1	5	− 0.76	0.03
The nurses’ provision of recommendations for care outside the dialysis centre	3.7	1.1	4	1	5	− 0.64	− 0.3
Overall nursing care at your dialysis centre	3.92	1.07	4	1	5	− 0.83	0.06

*M* Mean, *Me* Median, *SD* Standard deviation, *SKEW* Skewness coefficient, *KURT* Kurtosis coefficient, *Min *minimum, *Max *maximum

The KMO measure (0.96) and Bartlett’s sphericity test (χ2 = 2371.27; df = 136; *p* < 0.001) indicated the validity of the factor solution. The descriptive criterion and eigenvalues indicated a single-factor solution. The first factor exhibited an Eigenvalue of 13.512, while the possible second factor demonstrated an Eigenvalue lower than 1, with a value of 0.635. The single-factor solution accounted for 78% of the variance in the questionnaire results.

Table 2 presents the factor loadings calculated for individual components of the questionnaire.

**Table 2:** Factor loadings of individual components of the author’s questionnaire

Questionaire component	Loadings
The efficiency of nurses in performing their work	0.847
The presence of a nurse nearby when needed	0.804
The nurses’ knowledge of your illness and care	0.910
The speed with which nurses responded to your call/alarm	0.895
The way nurses created a friendly atmosphere during the procedure	0.792
The amount of information you received from nurses about your health and the scope of care	0.891
The frequency with which nurses checked to see if you were okay	0.849
The nurses’ willingness to help	0.912
The way the nurses explained various issues related to their actions to you	0.901
Your opinion on how the nurse treated you	0.910
How the nurses listened to your concerns and worries	0.866
The nurses’ willingness to fulfil your requests	0.934
The nurses’ assurance of privacy	0.918
The nurses’ awareness of your needs	0.869
The nurses’ provision of recommendations for care outside the dialysis centre	0.912
Overall nursing care at your dialysis centre	0.884
The efficiency of nurses in performing their work	0.927

It was evident that all factor loadings were elevated. It is evident that not a single one of the proposed questions was excluded. Cronbach’s alpha coefficient indicated that the scale constructed in this way is characterised by high reliability (0.983). Pearson’s correlation coefficient indicated a relatively strong relationship between the author’s scale for measuring satisfaction with nursing care and the KDQOL-SF scales – support from dialysis station staff (*r* = 0.541; *p* < 0.001) and satisfaction with nursing care at the dialysis station (*r* = 0.595; *p* < 0.001). This indicates an acceptable level of accuracy of the author’s tool.

The factor analysis indicated the possibility of reducing the dimensions of the tool to a single scale, which was provisionally named ‘satisfaction with nursing care’. The analysis of Cronbach’s alpha and Pearson’s r coefficients indicated that the constructed tool is characterised by high reliability and acceptable validity. The instrument was not subject to modification during the subsequent phases of the study.

### Main stage of the study

The main stage of the study comprised 202 haemodialysis patients, of whom 51.98% (105 patients) were female and 48.02% (97 patients) were male. The mean age of subjects in the study group was 52.78 years, with a standard deviation of 16.52 years, while the median age was 51 years. The age of the patients ranged from 21 to 92 years. The mean duration of dialysis treatment was 5.87 years, with a standard deviation of 8.75 years, and the median duration was 2.67 years. The shortest duration of dialysis was less than one month, and the longest was over 53 years. The most prevalent types of vascular access among patients were arteriovenous fistulas from their own vessels (exceeding 50% of the patient population) and tunnelled central venous catheters. The characteristics of the patients are presented in Table 3.

**Table 3:** Characteristics of patients

Variable		*n*	%	M	SD	Me	Min	Max
Age		202	**–**	52.78	16.52	51	21	92
Sex	Men	105	51.98					
	Female	97	48.02					
Type of vascular access	Arteriovenous Fistula (AVF)	134	66.34					
	Tunneled Central Venous Catheter (CVC)	58	28.71					
	Non-Tunneled Central Venous Catheter (CVC)	5	2.48					
	Arteriovenous Graft (AVG)	5	2.48					

*N* number of observations, % percentage, *M *mean, *Me* median, *SD* standard deviation; Min—minimum; Max—maximum.

The severity of vascular access problems was assessed using the VAQ questionnaire. In the analysed group of haemodialysis patients, the mean severity score was 13.79, with a standard deviation of 11.22, and the median score was 11. The present group exhibited a wide spectrum of vascular access challenges, ranging from minimal to severe, with a scale ranging from 0 to 62 points.

The Shapiro–Wilk test revealed a statistically significant deviation of the distribution of results from the normal distribution (W = 0.892; p = 0.000). The results obtained from the study indicate that the patients had a moderately low severity of vascular access problems. The detailed data can be found in Table 4.

**Table 4:** Severity of vascular access problems

Variable	n	M	SD	Me	Min	Max	Shapiro – Wilk Test	
							S-W	*p*
VAQ score	202	13.79	11.22	11	0	62	0.892	0.000

*N* number of observations, *M* mean, *Me* median, *SD* standard deviation, *S-W* Shapiro–Wilk test result, *p* test probability

The following section presents a statistical description of the results obtained from the study group for the purpose of examining the quality of nursing care. The study group was surveyed using a proprietary questionnaire. Furthermore, the relationships between satisfaction with nursing care and the severity of vascular access problems and pain during vascular access care were verified.

As illustrated in Table 5, the results of the original questionnaire on satisfaction with nursing care are presented.

**Table 5:** Satisfaction with nursing care – results of the author’s questionnaire

Variable	*n*	M	SD	Me	Min	Max	Shapiro – Wilk Test	
							S-W	*p*
Satisfaction with nursing care	202	3.79	1	4	1	5	0.928	0.000

*N* number of observations, *M* mean, *Me* median, *SD* standard deviation, *S-W* Shapiro–Wilk test result, *p* test probability.

In the study group of haemodialysis patients, the mean nursing care score was 3.79, with a standard deviation of 1.00 and a median of 4.00. The results obtained ranged from 1.00 to 5.00. The skewness and kurtosis statistics indicated that the distribution of nursing care ratings in this group exhibited slight left-skewness (with a predominance of high scores) and a normal distribution-like concentration of scores around the mean (SKEW = − 0.620; KURT − 0.417). The Shapiro–Wilk test demonstrated statistically significant disparities between the distribution of nursing care assessments obtained in the study sample and the normal distribution (*W* = 0.928; *p* = 0.000).

As illustrated in Table 6, a correlation exists between the satisfaction with nursing care and the severity of vascular access problems.

**Table 6:** Relationship between satisfaction with nursing care and severity of vascular access problems

Variable	VAQ score	
	*r*	*p*
Satisfaction with nursing care	− 0.387	0.000

*R* Pearson’s correlation coefficient r, *p* test probability.

A moderate correlation was identified between the severity of vascular access problems (VAQ score) and the assessment of nursing care (*r* = – 0.387; *p* = 0.000). It was found that patients who experienced more severe problems were less satisfied with the nursing care they received.

As illustrated in Table 7, a positive correlation exists between satisfaction with nursing care and the level of pain experienced.

**Table 7:** Relationship between satisfaction with nursing care and perceived pain level

Variable	Perceived pain	
	*r*	*p*
Satisfaction with nursing care	− 0.091	0.215

*R* Pearson’s correlation coefficient r, p test probability

The analysis did not reveal a statistically significant relationship between pain levels and satisfaction with nursing care (*r* = − 0.091; p = 0.215).

## Discussion

### Vascular access and satisfaction with nursing care

Patient satisfaction is widely regarded as a significant metric for evaluating healthcare outcomes, whilst concurrently exerting an influence on the clinical aspects of care. Furthermore, the perception of care can serve as a highly effective indicator for measuring patient well-being and the effectiveness of the therapeutic team, including nurses. The majority of studies on vascular access for haemodialysis employ objective clinical data, such as mortality, cardiovascular events and dialysis outcomes, as endpoints. While data from the perspective of patient care is of paramount importance, patients’ personal preferences, as expressed through satisfaction or dissatisfaction with care, are equally important to the patients themselves. A discordance between clinical data and patients’ personal preferences in terms of patient care priorities may be a factor contributing to diminished satisfaction and the failure to achieve the objectives of patient-centred care. Consequently, it is recommended that an assessment be conducted to ascertain patients’ personal preferences regarding nursing care, including care related to vascular access for haemodialysis [10–13].

Research conducted on groups of adult patients with End Stage Renal Disease (ESRD) undergoing dialysis has indicated the necessity to assess patient perception and satisfaction in the care planning process. In our own research, we utilised a proprietary questionnaire to measure satisfaction with nursing care in dialysis stations. In 2014, Palmer, de Berardis, Craig et al. conducted a study to assess the experiences of patients related to dialysis care. The study involved a sample of 2748 patients treated at Diaverum dialysis centres across a number of countries, including Hungary, Italy, Poland, Portugal and Argentina, among others. This study, in conjunction with data from other studies and our own, suggests that dialysis patients are, in the majority of cases, satisfied with the overall care provided at dialysis centres, including nursing care. However, a detailed analysis indicates a need for further evaluation and improvement of overall care, particularly in the area of education and information about the actions taken by staff, where the satisfaction rating obtained by the above-mentioned researchers was the lowest [4, 14, 15].

The objective of the present study was to ascertain the correlation between the functionality of vascular access for haemodialysis and patient satisfaction with care. Despite the moderate statistical correlation (*r* = − 0.387), the clinical significance is high. The findings suggest the presence of a discernible and patient-perceived relationship, whereby any escalation in the severity of vascular access problems is associated with a perceptible decline in satisfaction with a pivotal component of therapy, namely nursing care. Patients with a higher number of problems and a poorer quality of vascular access were found to be less satisfied with the quality of medical care provided. The discourse with patients during the collection of research material corroborated these relationships. Converging conclusions were reported by Taylor J. M. et al. in a study published in 2015 in Hemodialysis International. The present study constitutes a qualitative investigation, the objective of which was to assess the perception of vascular access functioning by 26 dialysis patients through the medium of structured interviews. The respondents indicated that they paid particular attention to aspects such as the aesthetics of vascular access, the fear of cannulation, dependence on others, the occurrence of problems, and fears about the future. The presence of a nurse during haemodialysis, where vascular access is utilised, has been identified by other researchers as being of significant importance to patients, enhancing the quality and satisfaction of care. In addition to paying close attention to the patient’s perspective, it is imperative to encourage their involvement and participation in their own care. This helps nurses to avoid adopting a purely ‘controlling’ attitude towards the patient. This relationship is likely multifactorial: access-related issues, such as the fear of cannulation or problems reported by Taylor et al., directly impact the patient-nurse interaction. Repeated difficulties can lead to patient frustration and a perception of care as less effective, which in turn erodes trust and lowers overall satisfaction. The findings of the aforementioned evaluations, in conjunction with the outcomes of our own investigative endeavours, illuminate domains in which vascular access care exerts a pronounced influence on patient perceptions and potentially delineates future trajectories for the enhancement of nursing care quality for vascular access to haemodialysis [15–20].

### Perceived pain and care of haemodialysis vascular access

Research conducted in the field of pain clearly demonstrates its impact on the overall well-being of patients – in physical, mental and social terms, and thus on their quality of life. Consequently, the evaluation of the occurrence and intensity of pain, in addition to its clinical benefits, should be incorporated into the patient’s psychological assessment. This approach has been demonstrated to enhance the efficacy of therapeutic interventions in cases where patients experience difficulty in identifying their pain (e.g., when pain is a manifestation of masked depression), in instances of treatment resistance, or when the patient exhibits a lack of cooperation [21, 22].

The present study also sought to assess the level of pain experienced by patients during direct nursing care of vascular access. The pain associated with access care is a component of problems related to the functioning of vascular access. It is a salient feature of the pre-dialysis patient population that they experience a heightened sense of trepidation with regard to the potential discomfort of the procedure. The findings of our research, which was conducted using a visual analogue scale (VAS) for pain assessment, demonstrated that the majority of patients exhibited low pain intensity during vascular access care. The mean pain level, measured on an 11-point scale, was 2.69. Research conducted by other scholars yielded analogous results. For instance, G.Çelik et al. (VAS 2.88) and A.A. Ghods et al. employed the numerical rating scale (NRS), yielding an average score of 2.91. In addition, the researchers evaluated the impact of pharmacological interventions designed to alleviate pain, including creams containing lidocaine or prilocaine, and non-pharmacological approaches such as cooling sprays, essential oils, physical activity, music therapy, and distraction techniques. Despite the evidence from the cited studies demonstrating the efficacy of the aforementioned methods in reducing pain associated with vascular access care, their utilisation does not appear to be wholly essential in routine practice for patients exhibiting low baseline pain levels [23–27].

### Limitations of the study

It is imperative to acknowledge the limitations imposed by the conducted research. The selection of the study group could be expanded to include patients treated in dialysis centres in other countries, which would increase the representativeness of the study. Moreover, the aforementioned analysis fails to consider objective factors associated with vascular access functionality, including blood flow in the arteriovenous fistula, laboratory test results, and the adequacy of dialysis using access, as measured by the Kt/V index. The incorporation of the aforementioned clinical variables and separate analysis for patients with different vascular access types should be considered when conducting further studies in this area. It should also be emphasized that the cross-sectional nature of the study allows for the identification of statistical relationships but does not permit conclusions about a cause-and-effect relationship between the analyzed variables. At the same time, this study design was deliberately chosen to meet the primary objective: capturing the relationship between a patient’s subjective perception and their satisfaction. The strength of this approach lies in providing a snapshot of the patient’s subjective reality, which is a crucial, and often overlooked, indicator of quality of care.

## Conclussions

The study demonstrated the importance of non-clinical aspects affecting the functioning of vascular access for haemodialysis, in particular:

1. The functionality of vascular access has been demonstrated to have a direct impact on the satisfaction levels of patients receiving nursing care. The greater the severity of vascular access problems experienced by haemodialysis patients, the lower the level of satisfaction with care. This highlights the need for multidirectional actions by nursing staff to maintain and improve the function of vascular access for haemodialysis.

2. In patients exhibiting low pain intensity during vascular access care, the level of satisfaction with nursing care remains unaffected. It is recommended that research investigating this impact in patients with significantly higher pain levels be expanded.

## Data Availability

No datasets were generated or analysed during the current study.
